# Implementation of a novel *in vitro* model of infection of reconstituted human epithelium for expression of virulence genes in methicillin-resistant *Staphylococcus aureus* strains isolated from catheter-related infections in Mexico

**DOI:** 10.1186/1476-0711-13-6

**Published:** 2014-01-09

**Authors:** Gloria Luz Paniagua-Contreras, Eric Monroy-Pérez, Felipe Vaca-Paniagua, José Raymundo Rodríguez-Moctezuma, Erasmo Negrete-Abascal, Sergio Vaca

**Affiliations:** 1 FES-Iztacala, Universidad Nacional Autónoma de México, Av. de Los Barrios 1, Los Reyes Iztacala, Tlalnepantla, 54090, Edo. de México, México; 2 Instituto Nacional de Cancerología, Laboratorio de Genómica, México, DF, México; 3 Instituto Mexicano del Seguro Social, G. Baz esq. F. Gómez, Tlalnepantla, Estado de México, México

**Keywords:** MRSA, Haemodialysis catheter, Virulence factors

## Abstract

**Background:**

Methicillin-resistant *Staphylococcus aureus* (MRSA) are clinically relevant pathogens that cause severe catheter-related nosocomial infections driven by several virulence factors.

**Methods:**

We implemented a novel model of infection *in vitro* of reconstituted human epithelium (RHE) to analyze the expression patterns of virulence genes in 21 MRSA strains isolated from catheter-related infections in Mexican patients undergoing haemodialysis. We also determined the phenotypic and genotypic co-occurrence of antibiotic- and disinfectant-resistance traits in the *S. aureus* strains, which were also analysed by pulsed-field-gel electrophoresis (PFGE).

**Results:**

In this study, MRSA strains isolated from haemodialysis catheter-related infections expressed virulence markers that mediate adhesion to, and invasion of, RHE. The most frequent pattern of expression (present in 47.6% of the strains) was as follows: *fnbA, fnbB, spa, clfA, clfB, cna, bbp, ebps, eap, sdrC, sdrD, sdrE, efb, icaA,* and *agr*. Seventy-one percent of the strains harboured the antibiotic- and disinfectant-resistance genes *ermA*, *ermB, tet(M), tet(K), blaZ, qacA*, *qacB,* and *qacC.* PFGE of the isolated MRSA revealed three identical strains and two pairs of identical strains. The strains with identical PFGE patterns showed the same phenotypes and genotypes, including the same *spa* type (t895), suggesting hospital personnel manipulating the haemodialysis equipment could be the source of catheter contamination.

**Conclusion:**

These findings help define the prevalence of MRSA virulence factors in catheter-related infections. Some of the products of the expressed genes that we detected in this work may serve as potential antigens for inclusion in a vaccine for the prevention of MRSA-catheter-related infections.

## Introduction

*Staphylococcus aureus* is a bacterial pathogen that causes multiple infections in humans, ranging from superficial skin infections to endocarditis, bone and joint infections, septic shock [1], and severe haemodialysis catheter-related infections [2]. *S. aureus* produce a broad spectrum of extracellular and cell wall-associated virulence determinants that contribute to the severity of infection [3]. Microbial adherence to cells and extracellular matrix is an essential first step in the process of colonization and infection [4], for which *S. aureus* express numerous surface adhesins known as microbial surface components recognizing adhesive matrix molecules (MSCRAMMs). These adhesins mediate adherence to host extracellular matrix components such as fibrinogen, fibronectin, and collagen [5]. Pathogenic adhesins include Fibronectin-binding protein A and B (FnBPA and FnBPB); *Staphylococcus* protein A (Spa); clumping factor A (ClfA); clumping factor B (ClfB); collagen adhesion (Cna); sialoprotein-binding protein (Bbp); elastin-binding protein of *Staphylococcus aureus* (EbpS); extracellular adhesion protein (Eap); serine aspartate repeat proteins C, D, and E (SdrC, SdrD, and SdrE; [3]); and extracellular fibrinogen-binding protein (Efb; [6])*.* Furthermore, pathogenic *S. aureus* strains exhibit a great capacity for biofilm formation on surfaces, making endovascular catheters a favourable niche for infection. Biofilm formation requires synthesis of PNAG (polymeric N-acetylglucosamine); the enzymes responsible for its synthesis are encoded by the *icaADBC* operon [7]. Expression of most virulence factors in *S. aureus* is controlled by the *agr* locus [8].

The number of methicillin-resistant *S. aureus* (MRSA) strains has steadily increased and nosocomial infections caused by MRSA have become a serious problem worldwide, as its presence has dramatically reduced the number of effective antibiotics available for the prevention and treatment of infections in hospitals and communities [9]. The main mechanism of methicillin resistance involves expression of the *mecA* gene, which encodes penicillin-binding protein 2a (PBP2a), a transpeptidase with low affinity for β-lactams [10]. MRSA strains frequently carry genes for multidrug resistance such as *blaZ*, which codes *β−*lactamases and confers resistance to *β*-lactams; *ermA, ermB*, and *ermC* confer erythromycin resistance; *aac(6*9*)-Ie-aph(2*0*)-Ia* confers aminoglycoside resistance; *tet(M), tet(O)*, and *tet(K)* confer resistance to tetracycline; and *vanA* and *vanB* confer vancomycin resistance [11]. Widespread use of quaternary ammonium compounds (QAC) in hospitals contributes to the selection of disinfectant-resistant *S. aureus*[12]. In several staphylococcal species, the *qacA, qacB*, and *qacC* genes have been identified in plasmids that also contain antibiotic-resistance genes [13-15].

The expression of virulence factors of *S. aureus* has been studied *in vivo* in animal models of infection [16,17], but immune cellular factors and nutritional conditions have affected the expression of virulence determinants. To circumvent these problems, in this study we propose a novel model of infection *in vitro* of reconstituted human epithelium (RHE) to analyze the expression patterns of virulence genotypes of MRSA strains isolated from catheter-related infections in Mexican patients undergoing haemodialysis. We also determined the phenotypic and genotypic combinations of antibiotic- and disinfectant-resistance in the *S. aureus* strains, which also were analysed by pulsed-field-gel electrophoresis (PFGE).

## Materials and methods

### Bacterial strains

Twenty-one *S. aureus* strains were donated by the Laboratorio Clínico de la Facultad de Estudios Superiores Iztacala (Universidad Nacional Autónoma de México) for phenotypic and molecular analysis. These strains were isolated from catheter-related infections of ambulatory patients (n = 21) undergoing treatment at a public hospital in Estado de México, México from January to May 2013. The bacterial strains were identified by classical microbiologic methods: Gram staining; mannitol, catalase, and coagulase activity (Bactident-coagulase, Merck); and the Api 32 Staph test (BioMerieux). *S. aureus* strains were molecularly characterized by PCR amplification of 23S rRNA, coagulase (*coa*), thermonuclease (*nuc*), clumping factor (*clf*A), protein A region X (*spa*), *femA*, and *femB*[18,19]. Bacterial DNA was extracted with the Wizard Genomic DNA Purification Kit (Promega). Resistance to methicillin was determined by the cefoxitin disc diffusion test (Becton Dickinson; inhibition zone, ≤21 mm) [20]. β-Lactamase enzymes were detected by using paper discs impregnated with the chromogenic cephalosporin nitrocephin (Becton Dickinson, USA). This substrate changes from yellow to red after the amide bond of the β-lactam ring is hydrolysed by β-lactamase. The change in colour was observed from 5 min–1 h. The *mecA* gene, which encodes methicillin resistance, was identified by conventional PCR [21]. *S. aureus* ATCC 25923 (*mecA* -) and ATCC 33592 (*mecA*+) were used as controls in each test.

### Antibiotic susceptibility testing

The standard disc diffusion method of Kirby-Bauer in Mueller Hinton agar (Bioxon) was used to evaluate antibiotic susceptibility. Multidiscs for Gram-positive bacteria were used (Bio-Rad). Results were interpreted in accordance with Clinical and Laboratory Standards Institute guidelines [20]. The antibiotics were ampicillin (AM, 10 μg), cefalotin (CF, 30 μg), cefotaxime (CTX, 30 μg), levofloxacin (LEV, 5 μg), cefuroxime (CXM, 30 μg), dicloxacillin (DC, 1 μg), erythromycin (E, 15 μg), gentamycin (GE, 10 μg), cefepime (FEP, 30 μg), penicillin (PE, 10 U), tetracycline (TE, 30 μg), and trimethoprim-sulfamethoxazole (SXT, 25 μg). *S. aureus* ATCC 25923 (*mecA*-) and ATCC 33592 (*mecA*+) strains were used as controls in each test.

### Detection of antibiotic and disinfectant resistance genes

The *tet(M), tet(O), tet(K), vanA*, *vanB*, *aac(6*9*)-Ie-aph(2*0*)-Ia* and *blaZ* genes were identified by PCR as described by Rizzotti *et al.*[11] and *ermA, ermB, ermC, msrA, mef, qacA, qacB*, and *qacC* as described by Zmantar *et al.*[12].

### SCC*mec* types and *agr* groups

A multiplex PCR with four primer pairs was used to identify the five main known SCC*mec* types [22] and another multiplex PCR with five primers was used to identify the *agr* groups [23].

### RHE inoculation with *S. aureus*

Reconstituted human epithelium (RHE; SkinEthic Laboratories, Nice, France) consists of human epithelial cell lines cultured on polycarbonate filters *in vitro* at the air-liquid interface in serum-free chemically defined medium. A total of 2 × 10^6^ *S. aureus* cells suspended in 50 μL PBS were inoculated onto the surface of the RHE and incubated at 37°C for 72 h with 5% CO_2_ and saturated humidity. The maintenance media was changed every 24 h.

### *S. aureus* RNA purification and reverse transcription

*S. aureus* cells were harvested from the RHE and suspended in 1 mL RNA Protect Bacteria Reagent (Qiagen). The sample was vortexed 30 s and incubated at room temperature 5 min. After centrifugation at 9400 rpm for 10 min, the cells were resuspended in 200 μL TE buffer (10 mM Tris–HCl, 1 mM EDTA, pH 8) containing 10 mg/mL lysozyme and 40 mg/mL lysostaphin. The sample was vortexed 10 s and incubated at room temperature 5 min. Total RNA was purified with the RN easy Mini Kit (Qiagen) according to the manufacturer’s instructions, including DNase treatment. The concentration and purity of total RNA were analysed with a NanoDrop 2000 spectrophotometer (Thermo Scientific). cDNA synthesis was performed with the QuantiTec Reverse transcription kit (Qiagen) according to the manufacturer’s instructions.

### Real-Time PCR amplification

The primers for Real-Time PCR were described previously as follows: *fnbA, fnbB, spa, clfA*, *clfB, cna, bbp, ebpS, eap, SdrC, sdrD, sdrE,* and *efb*[3]; and *agr*[24]. The Rotor-Gene SYBR Green PCR kit (Qiagen) was used for Real-Time PCR expression profiling of *icaA*[25] and *gyrB* (reference gene; [26]) using a Rotor Gene Probe PCR Kit (Qiagen). *S. epidermidis* ATCC 35984 and *Escherichia coli* ATCC 11775 were used as negative controls. *S. aureus* ATCC 33592 was used as the positive control.

### PFGE typing

MRSA isolates were PFGE typed by preparation of DNA and resolution of the *SmaI*-digested fragments as described by McDougal *et al.*[27]. Samples were separated on a CHEF-DR II system (Bio-Rad). Gels were photographed and digitized using a Bio-Rad Gel Doc. PFGE patterns were analysed as described by Tenover *et al.*[28] for bacterial strain typing.

### Data analysis

PFGE patterns were analysed with Gene Tool and Gene Directory software (Syngene). Reference standard *S. aureus* NCTC 8325 was included in each gel for band normalization. Percent similarities were obtained from the weighted pair group with mathematical average (UPMGA) based on Dice coefficients. Band position tolerance was set at 1.25%. A similarity coefficient of 80% was selected to define the pulsed-field type clusters.

### *spa* typing

The polymorphic X region of the protein A coding gene (*spa*) was amplified and sequenced as described [29]. Corresponding *spa*-types were assigned using the SPA Searcher website (http://seqtools.com). Ridom *spa* types were subsequently assigned using the spa-typing website (http://www.spaserver.ridom.de/) developed by Ridom Gmb.

## Results

All *S. aureus* strains (n = 21) were resistant to methicillin (Table 1). All MRSA strains (n = 21) expressed 7/15 studied genes: *spa, clfB cna, bbp, sdrC, icaA,* and *agr*. [group II (n = 18); group I (n = 3)] during *in vitro* infection of RHE (Table 2); 95.2% (n = 20) expressed *sdrD* and *efb*; 90.4% (n = 19) expressed *fnbA, clfA, ebps*, and *eap;* 85.7% (n = 18) expressed *sdrE*; and 80.9% (n = 17) expressed *fnbB*.

**Table 1:** Antibiotic-resistance phenotypes and PCR detection of genes encoding antibiotic and disinfectant resistance in the MRSA strains

		**Antibiotic-resistance phenotypes**													**β- lact amase PROD**	**Antibiotic- resistance genotypes**												**Disinfectant-resistance genotypes**		
**Strain**	**SCC** ***mec***	**Cefoxitin (MRSA)**	**CF**	**LEV**	**E**	**AM**	**TE**	**SXT**	**CTX**	**GE**	**CXM**	**FEP**	**DC**	**PE**		***ermA***	***ermB***	***ermC***	***mrsA***	***mef***	***tet(M)***	***tet(O)***	***tet(K)***	***vanA***	***vanB***	***aac(6*** **9 *)Ie- aph(2 *** **0** ***)Ia***	***blaZ***	***qacA***	***qacB***	***qacC***
S-58	I	R	R	R	R	R	R	R	R	R	R	R	R	R	+	+	+	-	-	-	+	-	+	-	-	+	+	+	+	+
S-76		R	S	S	R	R	R	S	S	S	S	S	R	R	+	+	+	-	-	-	+	-	+	-	-	-	+	-	-	+
S-106		R	R	R	R	R	R	S	R	S	R	R	R	R	+	+	+	-	-	-	+	-	+	-	-	-	+	+	+	+
S-10	II	R	R	R	R	R	R	S	R	S	R	R	R	R	+	+	+	-	-	-	+	-	+	-	-	-	+	+	+	+
S-59		R	R	R	R	R	R	S	R	S	R	R	R	R	+	+	+	-	-	-	+	-	+	-	-	-	+	-	-	+
S-66		R	R	R	R	R	R	S	R	S	R	R	R	R	+	+	+	-	-	-	+	-	+	-	-	-	+	-	-	+
S-73		R	S	S	R	R	R	S	R	S	S	R	S	R	+	+	+	+	-	-	+	-	+	-	-	-	+	+	+	+
S-75		R	R	R	R	R	R	S	R	S	R	R	R	R	+	+	+	-	-	-	+	-	+	-	-	-	+	-	-	+
S-108		R	R	S	R	R	R	S	R	R	R	R	R	R	+	+	+	+	-	-	+	-	+	-	-	+	+	+	+	+
S-16	IV	R	S	S	R	R	R	S	R	S	S	S	R	R	+	+	+	-	-	-	+	-	+	-	-	-	+	-	-	+
S-19		R	R	R	R	R	R	S	R	R	R	R	R	R	+	+	+	-	-	-	+	-	+	-	-	+	+	+	+	+
S-22		R	R	R	R	R	R	R	R	S	R	R	R	R	+	+	+	-	-	-	+	-	+	-	-	-	+	+	+	+
S-36		R	R	R	R	R	R	R	R	S	R	R	R	R	+	+	+	-	-	-	+	-	+	-	-	-	+	+	+	+
S-52		R	R	R	R	R	R	S	R	S	R	R	R	R	+	+	+	-	-	-	+	-	+	-	-	-	+	+	+	+
S-77		R	S	S	R	R	R	S	R	S	S	R	R	R	+	+	+	-	-	-	+	-	+	-	-	-	+	+	+	+
S-79		R	S	S	R	R	R	S	R	S	S	R	R	R	+	+	+	-	-	-	+	-	+	-	-	-	+	+	+	+
S-82		R	S	S	R	R	R	S	R	S	S	R	R	R	+	+	+	-	-	-	+	-	+	-	-	-	+	+	+	+
S-93		R	S	S	R	R	R	S	R	S	R	R	S	R	+	+	+	-	-	-	+	-	+	-	-	-	+	+	+	+
S-103		R	R	R	R	R	R	S	R	S	R	R	R	R	+	+	+	-	-	-	+	-	+	-	-	-	+	+	+	+
S-107		R	S	S	R	R	R	S	R	S	S	R	R	R	+	+	+	-	-	-	+	-	+	-	-	-	+	+	+	+
S-8		R	R	R	R	R	R	S	R	S	R	R	S	R	+	+	+	-	-	-	+	-	+	-	-	-	+	+	+	+
Nº. of resistant strains		21	13	12	21	21	21	3	20	3	14	19	20	21	21	21	21	2	0	0	21	0	21	0	0	3	21	16	16	21

R= Resistant; S=Sensitive.

**Table 2:** **Expression of virulence genes, *spa *** **typing, and PFGE in the MRSA strains**

**Strain**	***fnbA***	***fnbB***	***spa***	***clfA***	***clfB***	***cna***	***bbp***	***ebps***	***eap***	***sdrC***	***sdrD***	***sdrE***	***efb***	***icaA***	***agr *** **Group**	***spa *** **typing**	*PFGE
S-10	+	+	+	+	+	+	+	+	+	+	+	+	+	+	II	t895	1^A^
S-16	+	+	+	+	+	+	+	+	+	+	+	+	+	+	II	t002	2^B^
S-19	+	+	+	+	+	+	+	+	+	+	+	-	+	+	II	t895	
S-22	+	+	+	+	+	+	+	+	+	+	+	+	+	+	II	t895	3^C^
S-36	+	+	+	+	+	+	+	+	+	+	+	+	+	+	II	t895	
S-52	-	+	+	-	+	+	+	+	+	+	+	+	+	+	II	t895	4^A^
S-58	+	+	+	-	+	+	+	+	+	+	+	-	+	+	II	t895	5^A^
S-59	+	+	+	+	+	+	+	+	+	+	+	+	+	+	II	t895	6^C^
S-66	+	+	+	+	+	+	+	+	+	+	+	+	+	+	II	t895	
S-73	-	+	+	+	+	+	+	+	+	+	+	+	+	+	II	t008	7^A^
S-75	+	-	+	+	+	+	+	+	+	+	+	+	+	+	I	t895	8^A^
S-76	+	-	+	+	+	+	+	+	+	+	+	+	+	+	II	t189	9^A^
S-77	+	+	+	+	+	+	+	+	+	+	+	+	+	+	II	t895	10^C^
S-79	+	+	+	+	+	+	+	+	+	+	+	+	+	+	II	t895	
S-82	+	+	+	+	+	+	+	+	+	+	+	+	+	+	II	t895	
S-93	+	+	+	+	+	+	+	-	+	+	+	+	+	+	II	t008	11^A^
S-103	+	+	+	+	+	+	+	+	-	+	+	+	+	+	I	t895	12^A^
S-106	+	-	+	+	+	+	+	-	+	+	+	+	+	+	II	t895	13^A^
S-107	+	-	+	+	+	+	+	+	-	+	-	+	-	+	I	t008	14^A^
S-8	+	+	+	+	+	+	+	+	+	+	+	-	+	+	II	t895	15^D^
S-108	+	+	+	+	+	+	+	+	+	+	+	+	+	+	II	t895	
No	19	17	21	19	21	21	21	19	19	21	20	18	20	21	21		
%	90.4	80.9	100	90.4	100	100	100	90.4	90.4	100	95.2	85.7	95.2	100	100		

*A = Different, B = Possibly related, C = Identical and D = Closely related.

Without considering the 7 genes expressed by all MRSA strains, ten distinct expression patterns of virulence markers were found during MRSA infection of RHE *in vitro* (Table 3). Pattern 1, formed by the other 8 studied genes (*fnbA, fnbB, clfA, ebps, eap, sdrD, sdrE, ebf*) was present in ten (n = 47.7%) MRSA isolates, whereas patterns 2 and 3, both composed of 7 genes each, were expressed by two strains. The other seven patterns were constituted by 4–7 genes and were expressed only by one MRSA strain (Table 3).

**Table 3:** *Patterns of gene expression in the MRSA strains

**Pattern Nº**	**Expressed genes of the MRSA strains (*n*** **= 21)**	**Nº of strains (%)**	**Nº of genes per pattern (*n*** **= 15) Nº.%**
1	*fnbA, fnbB, clfA, ebps, eap, sdrD, sdrE, efb*	10 (47.7)	15 (100)
2	*fnbA, clfA, ebps, eap, sdrD, sdrE, efb*	2 (9.5)	14 (93.3)
3	*fnbA, fnbB, clfA, ebps, eap, sdrD, efb*	2 (9.5)	14 (93.3)
4	*fnbB, clfA, ebps, eap, sdrD, sdrE, efb*	1 (4.7)	14 (93.3)
5	*fnbA, fnbB, clfA, eap, sdrD, sdrE, efb*	1 (4.7)	14 (93.3)
6	*fnbA, fnbB, clfA, ebps, sdrD, sdrE, efb*	1 (4.7)	14 (93.3)
7	*fnbB, ebps, eap, sdrD, sdrE, efb*	1 (4.7)	13 (86.6)
8	*fnbA, fnbB, ebps, eap, sdrD, efb*	1 (4.7)	13 (86.6)
9	*fnbA, clfA, eap, sdrD, sdrE, efb*	1 (4.7)	13 (86.6)
10	*fnbA, clfA, ebps, sdrE*	1 (4.7)	11 (73.3)

*Without considering the 7 genes expressed by all strains (*spa, clfb, cna, bbp, sdrC, icaA,* and *agr*). *fnbA/B*=Fibronectin-binding protein A/B; *clfA*=clumping factor A; *ebps*=elastin-binding protein; *eap*=extracellular adhesion protein; *sdrD/E*=Serin aspartate repeat protein D/E; *efb*=Extracellular fibrinogen binding protein.

PFGE analysis showed that MRSA strains were distributed in 15 distinct electrophoretic patterns (data not shown). MRSA strains S-22 and S-36 (Table 2), isolated from the catheters of different patients, have identical electrophoretic patterns (data not shown) and the same *spa* type as the S-59 and S-66 MRSA strains (*spa* type t895). Three strains isolated from different patients showed 100% similarity by PFGE (S-77, S-79, S-82; Table 2) and belonged to the same *spa* type t895. Two closely related strains (S-8 and S108) were isolated from the catheters of two different patients and two possibly related strains (S-16 and S-19) were isolated from two different patients. The strains with identical PFGE patterns (S-22 and S-36; S-59 and S-66; S-77, S-79 and S-82) showed the same phenotypes and genotypes (Table 1). The most frequent *spa* types were t895 (76.2%; n = 16) and t008 (14.2%; n = 3; Table 2).

All MRSA strains were resistant to erythromycin (E), ampicillin (AM), tetracycline (TE), and penicillin (PE; Table 1). The frequency of resistance to other antibiotics tested was: cefotaxime (CTX) 95.2%, n = 20; dicloxacillin (DC) 95.2%, n = 20; cefepime (FEP) 90.5%, n = 19; cefuroxime (CXM) 66.7%, n = 14; cefalotin (CF) 62%, n = 13; levofloxacin (LEV) 57.1%, n = 12; trimethoprim-sulfamethoxazole (SXT) 14.3%, n = 3; and gentamycin (GE) 14.3%, n = 3 (Table 1). All MRSA strains were β-lactamase producers (Table 1). The following patterns of antibiotic resistance were found: 5 antibiotics (1 strain), 6 antibiotics (2 strains), 7 antibiotics (5 strains), 9 antibiotics (1 strain), 10 antibiotics (8 strains), 11 antibiotics (3 strains), and 12 antibiotics (1 strain) (Table 1).

Type IV SCC*mec* was identified by PCR in 12 MRSA strains, whereas type II SCC*mec* was detected in 6 strains, and type I SCC*mec* in 3 strains (Table 1). The *ermA* and *ermB* genotypes were identified in all strains, whereas *ermC* was detected only in S-73. All strains carried the *tet(M)*, tet(K), and *blaZ* genes*.* The *aac(6*9*)-Ie- aph(2*0*)-Ia* gene was identified in gentamycin-resistant strains S-19, S-58, and S-108 (Table 2). The *mrsA, mef, tet(O), vanA*, and *vanB* antibiotic resistance genes were not identified in any of the MRSA strains.

Our evaluation of disinfectant resistance revealed 100% (n = 21) of the MRSA strains carried *qacC*, 76% (n = 16) carried *qacA*, and 76% (n = 16) carried *qacB*. Fifteen strains (71.4%) showed the same phenotype/genotype pattern: resistance to erythromycin, ampicillin, tetracycline, cefotaxime, penicillin, β-lactamase production/*ermA*, *ermB, tet(M), tet(K), blaZ, qacA*, *qacB,* and *qacC* positivity*.*

## Discussion

Haemodialysis patients who are infected with methicillin-resistant *Staphylococcus aureus* (MRSA) are considered to have healthcare-associated (HA) infections [30,31].

For colonization and infection, bacterial adhesion to host extracellular matrix components like fibrinogen, fibronectin, and collagen is essential [5]. Therefore, there has been a strong interest in studying the involvement of proteins of the MSCRAMMs family of *S. aureus* using *in vivo* and *in vitro* models of infection [32-35]. In this study we implemented a novel model of infection *in vitro* of reconstituted human epithelium (RHE) to analyze the expression patterns of the MSCRAMMs family adhesion genes, and *icaA* and *agr* in *S. aureus* strains isolated from catheter-related infections in Mexican patients subjected to haemodialysis. Our data show that most of the genes that we studied were expressed by MRSA after infection of RHE (Table 2), which reflects the pathogenic behaviour of these strains. We identified ten different patterns of expression (Table 3), of which pattern No. 1, represented by the 15 genes studied (*fnbA, fnbB, spa, clfA, clfB, cna, bbp, ebps, eap, sdrC, sdrD, sdrE, efb, icaA, agr*), was present in ten MRSA strains (47.7%; Table 3). These results show that during infection of RHE, the MRSA strains expressed 11 to 13 genes coding for bacterial surface proteins; *icaA*, which participates in biofilm formation [7]; and the *agr* locus, which is a global regulator of multiple virulence factors [8]. These findings are consistent with the notion that pathogenesis of most *S. aureus* infections cannot be explained by the action of an unique virulence factor, but by several distinct factors acting in concert during the infective process [36]. Cna has been associated with endocarditis [37] and keratitis [38]. Fibronectin-binding proteins mediate adherence to human airway epithelium [4]. Clumping factor A (ClfA) plays an antiphagocytic role in neutrophils and macrophages [39] and is necessary for infection/pathogenesis in *in vivo* models of arthritis, sepsis, and endocarditis [40,41]. Clumping factor B (ClfB) mediates respiratory infection and attachment to cytokeratins on nasal epithelial cells [42] and the role of protein A (Spa) in *S. aureus* virulence has been demonstrated in models of sepsis and pneumonia [43]. Although the precise role of Sdr adhesins in staphylococcal infection is unknown, a strong correlation between the *sdr* genes of *S. aureus* and certain diseases has been reported. There is a significantly increased prevalence of the *sdrE* gene in invasive *S. aureus* strains [44], in *S. aureus* strains responsible for osteomyelitis [45] and in *S. aureus* isolates responsible for bone infections [46]. A recent report noted that Eap is associated with invasive diseases [33].

Earlier findings suggested a possible relationship between particular *agr* groups with the capacity of MRSA to cause specific illnesses. The *agr* I and *agr* II strains are associated with suppurative infections; *agr* III is associated with toxic shock syndrome toxin (TSST-1) mediated disease, and *agr* IV is associated with exfoliative toxin- and impetigo-producing strains [47]. Consistent with these findings, the strains reported in this work were isolated from suppurative catheter-related infections, or initiating suppurative catheter-related infections, and carried *agr*II (85.7%, n = 18) or *agr* I (14.3%, n = 3; Table 2).

Most of the strains reported here carried the SCC*mec* type IV (57%, n = 12; Table 1), an allele initially considered to be a characteristic of community-acquired MRSA (CA-MRSA; [48]). However, MRSA infections in dialysis-dependent patients have been considered to be mainly HA (healthcare-associated) according to epidemiologic classifications [49], and there are several reports of MRSA strains harbouring SCC*mec* type IV in HA infections [30,31,50-52]. The increase in multiple drug-resistant (MDR) MRSA has become a major challenge for the treatment of infectious diseases caused by what are known as superbugs. Strikingly, all the strains reported here were multidrug resistant β-lactamase producers. They were resistant to 5 to 12 antibiotics. None of the strains was sensitive to erythromycin, ampicillin, tetracycline, or penicillin (Table 1). On the other hand, only three strains (14.3%) were resistant to sulfamethoxasole/Trimethoprim or gentamycin. The high frequency of resistance to these antibiotics may reflect the fact that physicians of the public health service in Mexico prescribe all of these antibiotics, which are considered essential drugs in this sector.

The most frequent combination of antibiotic- and disinfectant-resistance phenotype/genotype in the MRSA strains (71.4%, n = 15) was: resistance to erythromycin, ampicillin, tetracycline, cefotaxime, penicillin, and β-lactamase production in association with *ermA*, *ermB, tet(M), tet(K), blaz, qacA*, *qacB,* and *qacC* genotypes (Table 1). These strains are not only resistant to five antibiotics, but also harbour three of the six plasmid-encoded MDR efflux pumps which mediate resistance to several biocides such as antimicrobial organic cations, including intercalating dyes (e.g., acriflavine and ethidium bromide), and quaternary ammonium compounds (e.g., benzalkonium chloride; [53]). Staphylococcal strains resistant to disinfectant have been identified in clinical isolates of MRSA from China (62%; [54]), Taiwan (55.4%; [55]), and Hong Kong (42%; [56]). All the erythromycin-resistant MRSA strains reported here carried the *ermA* and *ermB* genes and two strains (S-73 and S-108) carried the *ermC* gene. The *msrA* and *mef* genes were not detected in either strain (Table 1). We did not find discordances between the presence of *erm* genes and phenotypic resistance to erythromycin, as has been reported in other studies [12,57]. The incidence of *ermA* genes in our MRSA strains (100%) is higher than those reported in erythromycin-resistant *S. aureus*: 7.7% in Tunisia [12] and 16% in Denmark [58].

All MRSA strains were resistant to tetracycline and possessed the *tet(M)* and *tet(K)* genes (Table 1). Tetracycline is an antibiotic commonly used in Mexico to treat human and animal bacterial infections, contributing to the selection and propagation of resistant strains. Tetracycline resistance in *S. aureus* is encoded by the pT181 plasmid [59] and the *tet (M)* gene can be found in combination with *ermB* in the Tn916 transposon. The simultaneous presence of these genes has been found frequently in enterococci, as well as in streptococci and staphylococci [60]. Consistent with this, all the strains studied here carried these two genes, suggesting the presence of these genetic elements in the MRSA we analysed.

The gene *aac(6*9*)-Ie-aph(2*0*)-Ia* was detected in three strains (S-19, S-58, and S-108) that were also resistant to gentamycin; the *vanA* and *vanB* genes were not detected in any MRSA strain.

The strains with identical PFGE patterns showed the same phenotypes, genotypes, and spa type, suggesting that hospital personnel manipulating the haemodialysis equipment could be the cause of catheter contamination by these strains.

Our results are relevant because they demonstrate that MRSA strains isolated from catheter-related infections in haemodialysis patients express several virulence markers involved in the adhesion and invasion of RHE. We also analysed the phenotypes and genotypes of antibiotics and disinfectant resistance. These results will help define the incidence of virulence factors in MRSA associated with catheter-related infections and improve therapies in haemodialysis patients. In addition, some of the products of the expressed genes that we detected in this work may serve as potential antigens for inclusion in a vaccine for the prevention of MRSA-catheter-related infections.

## Competing interests

The authors declare that they have no competing interests.

## Authors’ contributions

GLPC, EMP, JRRM and ENA have made substantial contributions to acquisition of data. FVP has made substantial contributions to analysis and interpretation of data. SV has been involved in drafting the manuscript and has given final approval of the version to be published. All authors have read and approved the manuscript.
